# Contamination Status and Risk Assessment of Paralytic Shellfish Toxins in Shellfish along the Coastal Areas of China

**DOI:** 10.3390/md22020064

**Published:** 2024-01-26

**Authors:** Guanchao Zheng, Xizhen Xu, Haiyan Wu, Liqiang Fan, Qianrui Wang, Jixing Peng, Mengmeng Guo, Dajin Yang, Zhijun Tan

**Affiliations:** 1 Key Laboratory of Testing and Evaluation for Aquatic Product Safety and Quality, Ministry of Agriculture and Rural Affairs, Yellow Sea Fisheries Research Institute, Chinese Academy of Fishery Sciences, Qingdao 266071, China; zhenggc@ysfri.ac.cn (G.Z.); xxz1821212@163.com (X.X.); liqiangfan91@gmail.com (L.F.);; 2 Chinese Academy of Agricultural Sciences, Beijing 100081, China; 3 China National Center for Food Safety Risk Assessment, Beijing 100000, China; wangqianrui@cfsa.net.cn (Q.W.); yangdajin@cfsa.net.cn (D.Y.); 4 State Key Laboratory of Mariculture Biobreeding and Sustainable Goods, Yellow Sea Fisheries Research Institute, Chinese Academy of Fishery Sciences, Qingdao 266071, China

**Keywords:** paralytic shellfish toxins, shellfish, liquid chromatography–tandem mass spectrometry (LC–MS/MS), risk assessment, China

## Abstract

Paralytic shellfish toxins (PSTs) are widely distributed in shellfish along the coast of China, causing a serious threat to consumer health; however, there is still a lack of large-scale systematic investigations and risk assessments. Herein, 641 shellfish samples were collected from March to November 2020, and the PSTs’ toxicity was detected via liquid chromatography–tandem mass spectrometry. Furthermore, the contamination status and potential dietary risks of PSTs were discussed. PSTs were detected in 241 shellfish samples with a detection rate of 37.60%. The average PST toxicities in mussels and ark shells were considerably higher than those in other shellfish. The PSTs mainly included N-sulfonylcarbamoyl toxins (class C) and carbamoyl toxins (class GTX), and the highest PST toxicity was 546.09 μg STX eq. kg^−1^. The PST toxicity in spring was significantly higher than those in summer and autumn (*p* < 0.05). Hebei Province had the highest average PST toxicity in spring. An acute exposure assessment showed that consumers in Hebei Province had a higher dietary risk, with mussels posing a significantly higher dietary risk to consumers. This research provides reference for the green and sustainable development of the shellfish industry and the establishment of a shellfish toxin prevention and control system.

## 1. Introduction

Over the past few decades, the incidence and intensity of harmful algal blooms (HABs) have increased globally, owing to rising ocean temperatures and increasing coastal eutrophication [1]. Some harmful algae can produce shellfish toxins, which can be classified according to the symptoms of poisoning as paralytic shellfish toxins (PSTs), amnesic shellfish toxins (ASTs), diarrhetic shellfish toxins (DSTs), and neurotoxic shellfish toxins (NSTs) [2,3]. Among them, PSTs are considered to be one of the algal toxins that can seriously endanger human health [4]. PST poisoning incidents account for approximately 64% of shellfish poisoning incidents, resulting in around 2000 cases per year globally [5,6]. PST poisoning occurs when shellfish contaminated with any of the STX-group analogs are ingested [7], and can be detected within minutes or hours [8]. Mild cases of PST poisoning present symptoms such as numbness of the lips and mouth, localized tingling sensations, paralysis of the body muscles, and fever, whereas severe cases can lead to complete paralysis and death from respiratory failure [6,9]. The mechanism of PSTs’ toxicity involves restraining the transduction of intracellular action potentials via the highly specialized inhibition of voltage-gated sodium channels [10]. PSTs are usually not toxic to shellfish themselves, but can be toxic to mammals by blocking cellular sodium channels [11]. PSTs are prevalent in marine and freshwater ecosystems throughout the world [12], and the rise in the occurrence of PST-induced poisoning is linked to the warming of oceans and the increase in HABs as a result of human activities [13]. Currently, the distribution and detection rates of PSTs continue to expand globally [14], posing a significant threat to human health.

PSTs possess a tetrahydropurine structure, and are primarily synthesized by dinoflagellates (*Alexandrium*, *Gymnodinium*, *Pyrodinium*, etc.) and blue-green algae in freshwater environments [15,16,17]. Currently, researchers have identified over 60 types of PST analogs [18], which can be divided into four categories based on the substituent group in R4 (Table S1): carbamate toxins, including saxitoxin (STX), neosaxitoxin (NEO), and gonyautoxin1-4 (GTX1-4); n-sulfocarbamoyl toxins, including GTX5, GTX6, and N-sulfocarbamoylgonyautoxin1-4 (C1-C4); decarbamoyl toxins, including decarbamoyl saxitoxin (dcSTX), decarbamoyl neosaxitoxin (dcNEO), and decarbamoyl gonyautoxin 1-4 (dcGTX1-4); and deoxydecarbamoyl toxins, including deoxydecarbamoyl saxitoxin (doSTX) and deoxydecarbamoyl gonyautoxin 2-3 (doGTX2-3). The filter-feeding characteristic of marine shellfish results in the accumulation of toxins within the soft tissues from the feeding of toxic algae [19], and the strong accumulation and biotransformation effect of PSTs in shellfish increases the risk of PST poisoning in humans consuming PST-contaminated shellfish. To manage the hazard of dietary poisoning caused by PSTs, the present international limit standard for PSTs in shellfish has been set at 800 μg STX eq. kg^−1^ by the European Food Safety Authority (EFSA) [20,21]. The acute exposure reference dose (ARfD) for PSTs established by the EFSA is 0.5 μg STX eq. kg^−1^ bw day^−1^ [22], while that set by the Food and Agriculture Organization of the United Nations (FAO), the Intergovernmental Oceanographic Commission (IOC), and the World Health Organization (WHO) is 0.7 μg STX eq. kg^−1^ bw day^−1^ [23].

China is a large aquaculture country, and its marine shellfish aquaculture industry ranks first in the world [24], with an annual marine shellfish production of about 1589 tons in 2022 [25]. Marine shellfish possess high protein, high mineral, and low fat contents, making them a healthy food option [26]. With the increase in the standard of living of Chinese consumers, the demand for high-quality protein sourced from shellfish is also rising. However, in recent years, *Alexandrium* and *Gymnodinium* HABs have frequently occurred in China’s offshore areas, mainly in the Bohai Sea and part of the nearshore sea areas in the East China Sea, resulting in recurrent PST poisoning incidents in many places [27]. The most prevalent microalgae that produce PSTs in the coastal waters of China are *Alexandrium* spp. [27], including *A. fundyense* in the Yellow Sea and Bohai Sea, *A. pacificum* in the waters south of the Yangtze River Estuary [28,29,30], and *G. catenatum,* mainly in the Bohai Sea, Lianyungang of Jiangsu Province, and the coastal waters of Fujian Province [27]. Based on a survey of China’s coastal areas, the overall rate of PSTs has been noted to be low and below the limits (800 μg STX eq. kg^−1^) in the Yellow Sea, East China Sea, and South China Sea [31]. However, the Bohai Rim showed a high detection rate of PSTs, reaching 98.0%, exceeding the limits by 12.0% [32], indicating a more severe contamination status. In 2016, severe shellfish poisoning occurred in Qinhuangdao, where the toxin levels surpassed the safety limit (800 μg STX eq. kg^−1^) by 65 times [33]. In recent years, there has been a rise in the poisoning incidents resulting from the consumption of toxic shellfish. For instance, from 1991 to 2003, 11 PST poisoning incidents were documented in Lianyungang City [34]. In addition, 164 people in Fujian Province were poisoned by consuming PST-contaminated shellfish [35]. PST contamination not only causes serious effects on human health, but also hinders China’s economic progress, warranting significant attention.

Currently, research on PSTs primarily includes small-scale investigations and risk assessments in specific areas [36,37], or monitoring long-term PST contamination in designated areas [38]. These studies have certain limitations and cannot fully and systematically reflect the overall distribution of PST contamination in China’s coastal areas and the dietary risk of consumers. Therefore, in the present study, a large-scale investigation of the distribution of 13 EFSA-restricted PST analogs of high concern was performed using liquid chromatography–mass spectrometry (LC-MS/MS). A total of 641 shellfish samples were obtained from aquaculture locations in six coastal provinces of China during the spring, summer, and autumn of 2020, and sequentially analyzed for PSTs. In addition, the potential risk of dietary exposure of consumers in coastal areas of China to PSTs through shellfish consumption was also assessed. The objectives of the present study were to (1) investigate the differences in the toxicities and profiles of PSTs in shellfish; (2) analyze the spatial and temporal distribution of PSTs and seasonal variations in different shellfish; and (3) evaluate the risk of dietary exposure of consumers in urban coastal areas of China to PSTs in shellfish.

## 2. Results

### 2.1. Difference in the Toxicities and Profiles of PSTs in Different Shellfish

A total of 14 shellfish species collected from offshore China were analyzed for 13 PSTs via LC-MS/MS. Table 1 shows the toxicities and detection rates of PSTs in the five groups of shellfish. The total overall detection rate was 37.60% the detection rates (>60.0%) of PSTs in mussels, scallops, and ark shells were significantly higher than those (≤30%) in clams and oysters. The average toxicity of PSTs in all the shellfish samples varied between 11.86 (LB) µg STX eq. kg^−1^ and 32.49 (UB) µg STX eq. kg^−1^. The highest range of average PST toxicity was found in ark shells, ranging from 62.23 (LB) to 94.67 (UB) µg STX eq. kg^−1^. Mussels presented the maximum PST toxicity, with their PST toxicity reaching up to 546.09 µg STX eq. kg^−1^ (LB)–576.02 µg STX eq. kg^−1^ (UB).

**Figure 1:**
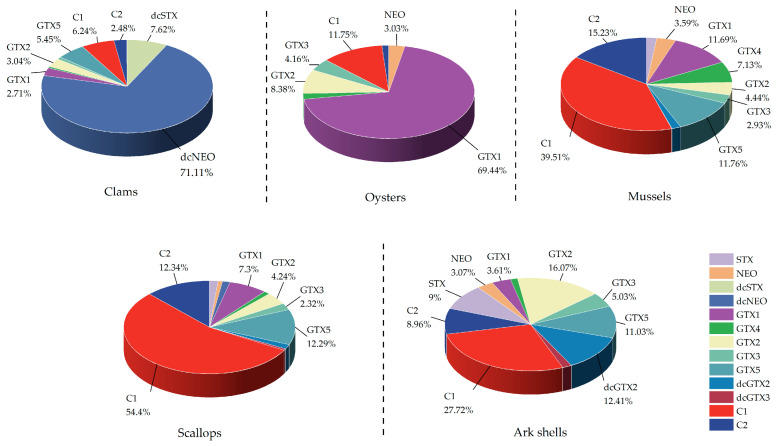
Percentage of different analogs of PSTs in five shellfish groups. The PST analogs within the five shellfish groups are summarized, with each analog’s proportion marked.

The percentages of the different PST analogs accumulated in the five shellfish groups are shown in Figure 1. The concentrations of the different PST analogs accumulated in the five shellfish groups considerably varied. Among them, the major toxins accumulated in clams and oysters were dcNEO and GTX1, respectively, with concentrations reaching more than 70%. The major PSTs accumulated in mussels and scallops were C1, C2, and GTX5, constituting more than 60% of the total PST analogs. In ark shells, the primary toxin was C1, with the other PST analogs presenting relatively low concentrations.

### 2.2. Seasonal Variation in PSTs’ Toxicities in Different Shellfish Groups

**Figure 2:**
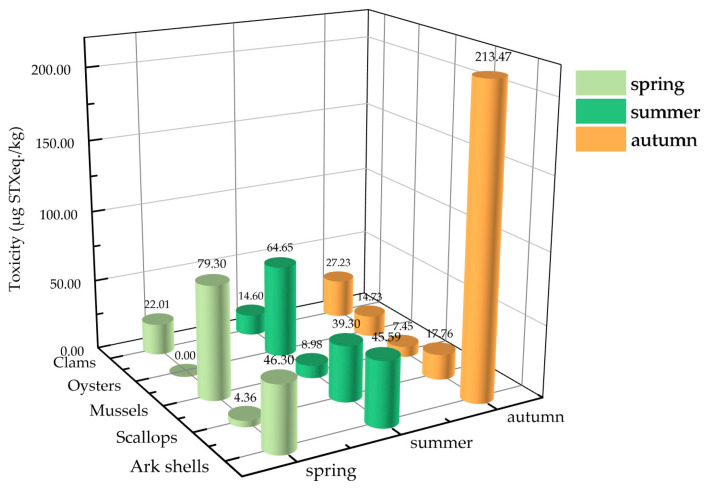
Seasonal differences in the average PST toxicities in different shellfish groups.

To determine the seasonal variation in the PSTs’ toxicities in the five shellfish groups, shellfish samples were collected across three seasons from March to November 2020, including spring (March–May), summer (June–August), and autumn (September–November). Figure 2 shows the seasonal variations in the average toxicities of the PSTs in the five shellfish groups. Significant differences were noted in the average toxicities of PSTs in shellfish in different seasons. Among them, mussels exhibited the highest average PST accumulation during spring (79.30 µg STX eq. kg^−1^), which was significantly higher than those recorded during summer and autumn. Oysters and scallops exhibited the highest average toxicity of PSTs (64.65 and 39.30 µg STX eq. kg^−1^, respectively) during summer, while clams and ark shells showed the highest average toxicity of PSTs in autumn (27.23 and 213.47 μg STX eq. kg^−1^, respectively). Overall, except for mussels, the toxicity of PSTs in most of the shellfish was significantly higher in summer and autumn.

### 2.3. Spatial and Temporal Variation in PSTs’ Toxicities in Shellfish Samples

**Figure 3:**
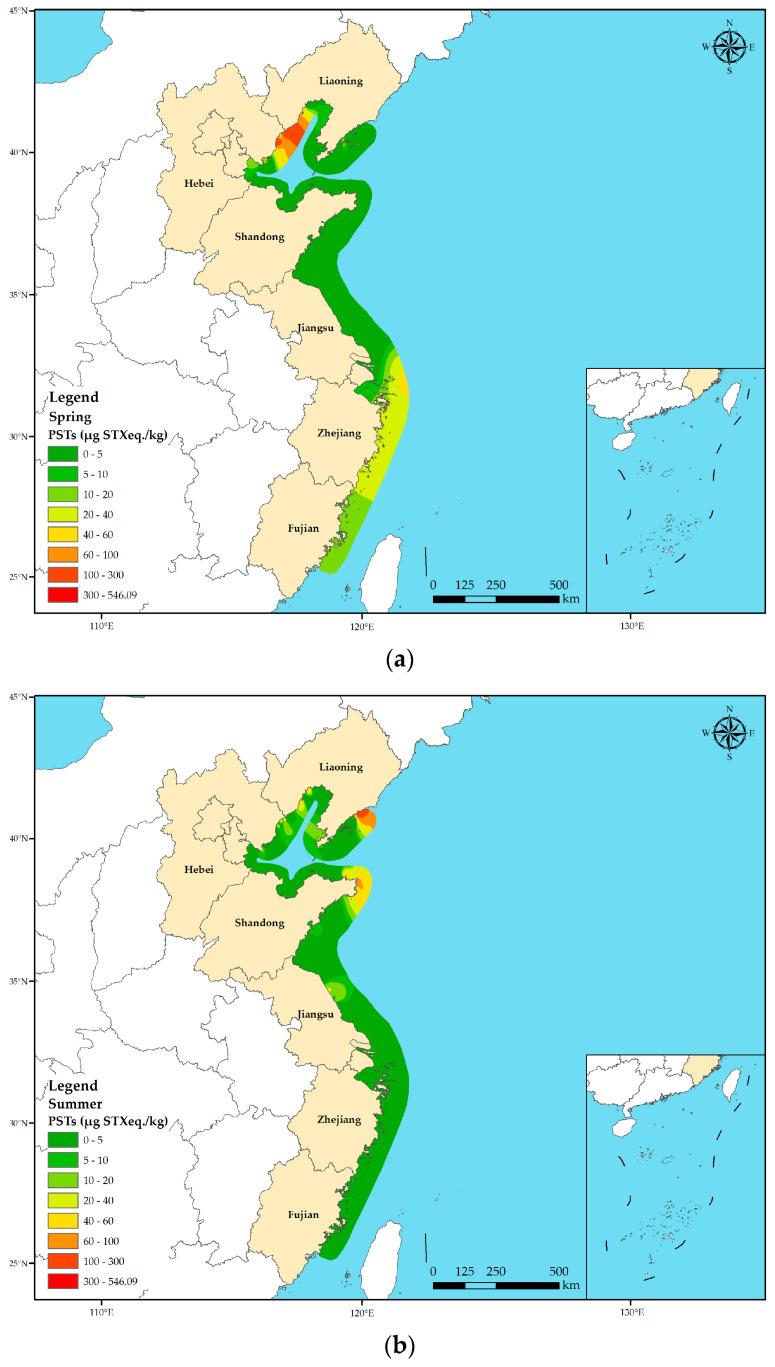

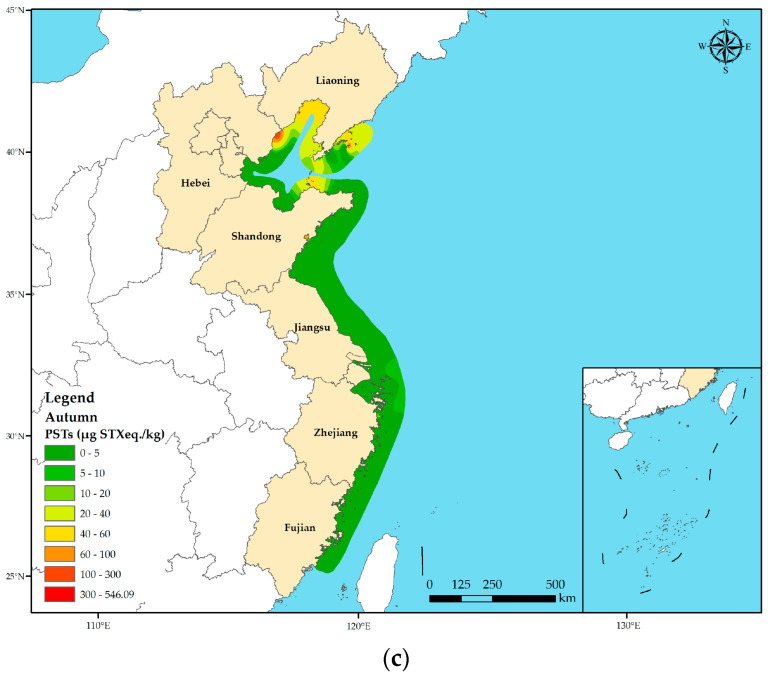
Seasonal variation in PST toxicity in shellfish in different coastal provinces of China. (**a**) Spring; (**b**) summer; (**c**) autumn. Based on the collected sample data, the PST toxicity distribution map across various seasons along the Chinese coast was generated using the spline function interpolation method through ArcGIS 10.8.

A total of 641 shellfish samples were collected from different provinces along the Chinese coast in three seasons. Overall, the average PST toxicity was notably higher in spring (22.45 µg STX eq. kg^−1^) when compared with that in summer (7.17 µg STX eq. kg^−1^) and autumn (10.22 µg STX eq. kg^−1^) across all shellfish species (*p* < 0.05) (Figure 3). However, there was no significant difference in the average PST toxicity in shellfish between summer and autumn. Furthermore, there were variations in the average PST toxicities across the provinces during different seasons. Hebei, Zhejiang, and Fujian Provinces presented maximum average PST toxicities in spring, with Hebei Province exhibiting the highest average PST toxicity of 546.09 µg STX eq. kg^−1^ in spring. The average PST toxicity in Shandong and Jiangsu Provinces was low in all the three seasons and did not exceed 10.00 µg STX eq kg^−1^. Furthermore, the average PST toxicity in Liaoning Province was the highest in autumn (30.77 µg STX eq. kg^−1^), which was significantly higher than those in spring and summer.

### 2.4. Acute Dietary Exposure Assessment

The acute dietary exposure of consumers in the coastal areas of China to PSTs was assessed based on the body weights and dietary intakes of shellfish in the exposed populations comprising different gender and age groups, as well as the PST residues in the shellfish samples collected (Table 2). The EDI (estimated daily intake) for different shellfish groups ranged from 0 to 0.78 µg STX eq. kg^−1^ bw day^−1^. Among the five shellfish groups, mussel consumption resulted in the highest dietary exposure to PSTs among all age groups, with a maximum value of 0.78 µg STX eq. kg^−1^ bw day^−1^. In terms of mussel consumption, the dietary exposure to PSTs for all age groups was less than 0.7 µg STX eq. kg^−1^ bw day^−1^, except for the 2–7-year age group and the group of males over 65 years of age. The consumption of the remaining four shellfish groups led to low dietary exposures to PSTs, ranging from 0 to 0.33 µg STX eq. kg^−1^ bw day^−1^.

**Figure 4:**
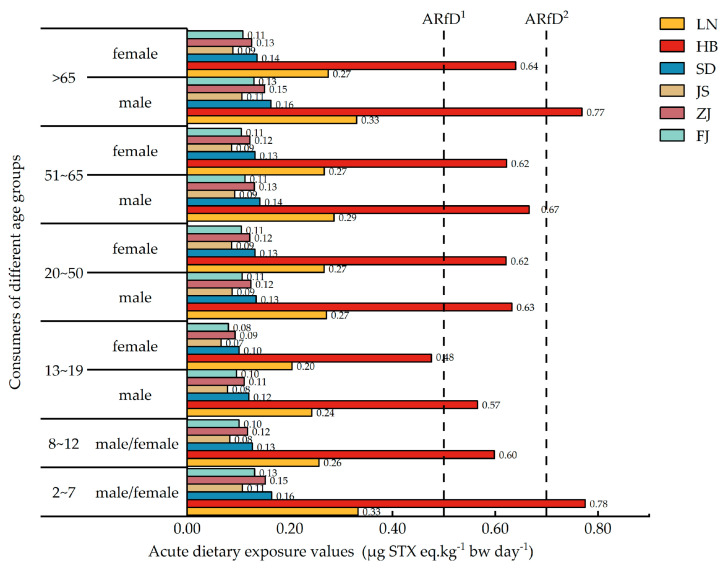
Acute dietary exposure of consumers in different coastal provinces of China to PSTs. ARfD^1^: 0.5 μg STX eq. kg^−1^ bw, formulated by EFSA; ARfD^2^: 0.7 μg STX eq. kg^−1^ bw, formulated by FAO/IOC/WHO. LN: Liaoning Province; HB: Hebei Province; SD: Shandong Province; JS: Jiangsu Province; ZJ: Zhejiang Province; FJ: Fujian Province.

Figure 4 shows the dietary exposure of consumers in different coastal provinces of China to PSTs, ranging from 0 to 0.78 µg STX eq·kg^−1^ bw day^−1^. The dietary exposure risk for consumers in Hebei Province was significantly higher than that for consumers in the other provinces, with children aged 2–7 years and elderly men aged over 65 years presenting the highest dietary exposure to PSTs. Except for consumers in Hebei and Liaoning Provinces, the dietary exposure of consumers in all the other provinces was low, ranging from 0 to 0.16 µg STX eq·kg^−1^ bw day^−1^. In addition, when compared with women, the dietary exposures of men to PSTs were generally higher across all age groups in different provinces.

## 3. Discussion

The widespread occurrence of HABs producing PSTs has raised concerns among global fisheries, aquaculture, and human health for several decades [39,40]. Although China has a vast coastline with abundant fishery resources, the presence of PST contamination in the seas has attracted attention. To establish a system for the prevention and control of PSTs in the coastal areas of China and reduce the risk of PST poisoning from shellfish consumption, investigation of coastal PST contamination and dietary risk assessment are necessary. In the present study, the PST detection rate considerably varied, and the toxicities and profile of PSTs in shellfish were significantly different among the five shellfish groups. Contamination with PSTs was prevalent among farmed shellfish off the Chinese coast with evident seasonal variance. During spring, mussels exhibited the highest average PST toxicity and the most severe contamination, thereby posing a dietary hazard to young children and the elderly.

There were significant differences in the toxicity of PSTs accumulated in different shellfish species. The average toxicities of PSTs in mussels and ark shells were considerably higher than those in oysters and clams, with mussels presenting the highest toxicity of accumulated PSTs. The overall accumulation rate of PSTs in mussels, as observed in the exposure experiment, reached 14.6%, which is higher than that found in oysters (8.3%) and Philippine clams (2.5%) [41]. Previous studies have also reported that *Mytilus edulis* and *Scapharca subcrenata* have a higher average PST toxicity than Pacific oysters [32], consistent with the results of the present study. The rate of toxin accumulation may account for the differences in the toxicity of PSTs in different shellfish. A previous study comparing the capability of oysters and mussels to accumulate and remove PSTs revealed that toxin accumulation in mussels was three-fold higher than that in oysters [42], highlighting the higher accumulation rate and toxicity of PSTs in mussels [43,44,45]. It must be noted that mussel neural axons are insensitive to the STX toxin of PSTs [46], whereas oysters are highly sensitive to PSTs [47]. Consequently, oysters accumulate lower levels of the toxin and swiftly release it [47]. Furthermore, differences in the rate of filter-feeding and the uptake of toxic algal cells by different shellfish can also lead to variations in the toxicity of PSTs in different shellfish. Mussels accumulate toxins quickly as a result of their high filtration rate during feeding [48]. Clams (80 µg per 100 g) have been reported to present a significantly lower rate of accumulation and maximum load of PSTs following exposure to *A. tamarense* when compared with mussels (1100 µg per 100 g), and the main toxin detected was C1/2 [49]. This could be due to the fact that mussels have a higher ingestion rate, whereas clams have a lower uptake rate and tend to produce pseudofeces when filtering large amounts of algal cells [50,51]. Moreover, the transcript levels of SODHSP70, catalase, ferritin, and various pattern recognition receptor genes involved in the immune response have been reported to significantly increase in mussels with increasing STX concentrations [52,53]. These findings may explain the high PST accumulations in mussels.

Furthermore, significant differences in the concentration of each analog of PSTs were detected in the five shellfish groups examined in this study. A previous work reported that the Chinese coastal shellfish primarily consisted of N-sulfonylcarbamoyl toxins (class C) and carbamoyl toxins (class GTX), accounting for over 50% of the total toxins present [54]. The current study noted that clams and oysters primarily accumulated dcNEO and GTX1 toxins, respectively, while mussels and scallops mainly accumulated C1, C2, and GTX5 toxins. This variation in the toxin accumulation and metabolic transformation abilities of different PST analogs may account for the significant differences in PST analogs in various shellfish. Additionally, the differences in the PST analogs in algae fed on by shellfish can also lead to variations in PST analogs in the shellfish. It has been reported that the main toxins found in *A. fundyense* and *A. catenulatum* in the Yellow Sea and Bohai Sea region were C1-2, GTX2-3, and GTX5 [55], which closely resemble the primary analogs of PSTs in mussels and scallops, as observed in the present study. A comparative research study on PST analogs in *A. tamarense* and mussels revealed that the major toxins present in *A. tamarense* were C2 and GTX4, and that the PST analogs in mussels during the early stages of exposure were similar to those found in the algal cells [56]. Furthermore, C1-2 and GTX1 and 4 have been noted to be the common major toxin analogs in *A. tamarense* and *M. edulis* [57]. It must be noted that PSTs also undergo transformation reactions during cumulative exposure in shellfish, and significant interspecific disparities in their biotransformation capacity have been recorded [58]. The enzymes and bacteria found in shellfish can facilitate the conversion of PSTs with varying structures and species, leading to distinct levels of PST analogs in shellfish [59]. For example, GTX4 was predominant in *A. tamarense*, while GTX1 was prominent in clams and oysters after the ingestion *of A. tamarense* [60]. Aminomethylhydrolase I, which was isolated and purified for the first time from the hepatopancreas of Chinese clams, has been observed to catalyze the hydrolysis of the R4 group of carbamates or N-sulfocarbamoyl groups to generate deaminomethyl toxins [61]. The conversion of N-sulfocarbamoyl to carbamate toxins has also been reported to be prevalent in shellfish, such as the conversion of class C to GTXs of PSTs in mussels exposed to *A. fundyense* [62]. Furthermore, shellfish bacteria can also facilitate the transformation of PSTs, and the bacteria in *M. edulis* could convert GTXs into the profoundly toxic STX through a reduction reaction [63].

The present study observed obvious regional and seasonal differences in PST contamination along the Chinese coastal areas, as well as seasonal differences in the average accumulation of PSTs in shellfish. The geographic variations in PST contamination levels could be primarily attributed to the differences in harmful algal species found in distinct marine regions [64,65]. China’s coastal area spans a large latitude range, resulting in significant temperature variations across different sea regions within the same season and between seasons. Consequently, the growth and disappearance patterns of various harmful algae significantly differed between these areas. The accumulation of toxins in shellfish and the growth of toxic algae are closely linked to temperature seasonality that is conducive to toxic algal blooms. As shellfish filter-feed more toxic algae during the early stages of algal blooms, they are more prone to accumulate higher levels of toxins. The results of the present study showed that PST contamination was mainly observed in spring, with higher PST levels detected in the Yellow Sea and Bohai Sea, and lower PST levels noted in the East China Sea. Previous works have shown that shellfish in the Yellow Sea have the highest levels of PST contamination in spring, followed by those in summer, autumn, and winter [19], similar to the results of the present study. This finding may be attributed to the fact that the spring water temperature in the Yellow Sea is suitable for the growth of toxin-producing algae. For example, the optimal temperature range for *A. tamarense* proliferation is 7.5 °C–9.0 °C [66], and the average temperature of spring water in the North Yellow Sea ranges from 7.5 °C to 11.0 °C, providing an ideal environment for the growth of these harmful algae [67], and thus resulting in the maximum total toxicity of PSTs in spring. In contrast, water temperatures of 4.3 °C–8.5 °C in winter and 23.3 °C–27.8 °C in summer are unfavorable for the growth of *A. tamarense* [68]. Similarly, the near-shore water temperature in the Bohai Sea during spring ranges from 2.8 °C to 14.3 °C, providing optimum conditions for the growth of PST-producing algae [32,69]. The PSTs found in the Qinhuangdao Sea during spring are mainly produced by *A. tamarense* that could thrive at temperatures between 10 °C and 13 °C [33,70]. The growth of *Prorocentrum* is more favorable in the East China Sea during spring, when compared with that in other seasons, because the water temperature in the East China Sea during May is around 25 °C [71], which is conducive for achieving the maximum proliferation rate of marine *Prorocentrum* [72]. In addition, this temperature range has also been noted to be favorable for the occurrence of the *Prorocentrum* HAB [73]. In summary, temperature-induced disparities in the PST-producing dominant algal species across diverse marine regions may contribute to the varied geographical distribution of PST contamination [74,75].

China has an extensive record of PST contamination offshore, and despite the total PSTs in shellfish remaining within the regulatory limit, consumers may still face a certain risk of dietary exposure to PSTs owing the significant variation in shellfish intake [76]. The present study assessed the dietary risks of various shellfish to consumers of all ages in different provinces along the Chinese coast. The results showed significant differences in the dietary exposures of consumers of different genders in each province to PSTs, with male consumers of all age groups presenting a higher dietary risk than female consumers. Furthermore, consumers in Hebei Province had a significantly higher dietary exposure to PSTs when compared with those in other Provinces, and mussels posed a significantly higher dietary risk to shellfish consumers than other shellfish. In the coastal regions of China, with the exception of mussels, the dietary exposure of other shellfish groups was below the EFSA-recommended ARfD of 0.5 μg STX eq. kg^−1^ bw day^−1^. Moreover, the consumption of mussels within Hebei Province posed lower dietary exposure risks than the ARfD recommended by the FAO/IOC/WHO (0.7 μg STX eq. kg^−1^ bw day^−1^) to consumers, except for 2–7-year age group and the group of males over 65 years of age. In a similar dietary exposure assessment conducted in a neighboring Asian country, Korea, mussels exhibited the highest toxicity of PSTs, with an ARfD of 0.30 µg STX eq. kg^−1^ bw day^−1^ [14], which is lower than that observed in the present study. The gender disparities in dietary risks among consumers of all ages may be attributed to the larger diets of men. Moreover, consumers of different ages may exhibit different sensitivities to PSTs. It must be noted that the epithelial cells of the gastrointestinal tract become thinner and more fragile with increasing age, thus raising the incidence of ulcers and sensitivity to toxins [77]. Currently, PSTs remain widespread in the nearshore waters of Qinhuangdao and are extensively distributed throughout seawater and marine organisms [78]. Therefore, the long-term monitoring and management of PSTs in priority areas should be strengthened. Furthermore, as the effects of PSTs on different populations can vary, more attention should be paid to fish farmers and islanders, whose dietary intake of aquatic foods is higher than that of the general population [79].

## 4. Materials and Methods

### 4.1. Sample Preparation

The survey period was from March to November 2020, and the samples were collected from shellfish culture areas in China’s coastal provinces, including Liaoning (LN), Hebei (HB), Shandong (SD), Jiangsu (JS), Zhejiang (ZJ), and Fujian (FJ) Provinces. A total of 641 diverse shellfish samples, including 318 clams (*Ruditapes philippinarum*, *Mercenaria mercenaria*, *Meretrix meretrix*, *Cyclina sinensis*, *Mactra chinensis*, and *M. veneriformis*), 80 oysters (*Crassostrea gigas* and *C. ariakensis*), 150 mussels (*Mytilus coruscus* and *M. galloprovincialis*), 81 scallops (*Patinopecten yessoensis* and *Chlamys farreri*), and 12 ark shells (*Scapharca subcrenata* and *Tegillarca granosa*), were collected at random from the local shellfish culture areas, washed with clean water, kept in portable icy incubators below 4 °C, and transported to the lab within 24 h. All the shellfish samples were homogenized at 24,000 rpm using a T18 basic Ultra-Turrax mixer (IKA, Königswinter, Germany) and stored at −20 °C before the LC-MS/MS analysis.

### 4.2. Chemicals and Reagents

Chromatography-grade formic acid (≥98.0%) and ammonium formate (≥97.0%) were purchased from Fluka, Buchs, Switzerland, and chromatography-grade ammonia and mass spectrometry-grade acetic acid were obtained from Sigma, Mo, USA. Mass spectrometry-grade acetonitrile was supplied by Merck, Darmstadt, USA. A graphitized carbon black solid-phase extraction column (ENVI-CarbTM, 250 mg/3 mL) was obtained from Supelco, Bellefonte, PA, USA. PST standards (STX, dcSTX, NEO, dcNEO, GTX1&GTX4, GTX2&GTX3, GTX5, dcGTX2&dcGTX3, C1&C2) were provided by the National Research Council of Canada (Halifax, Nova Scotia, Canada). Deionized water (18.2 MΩcm) was obtained using the Milli-Q system (Millipore, Bedford, MA, USA) equipped with ion-exchange and carbon filters.

### 4.3. PSTs Extraction

The PSTs were analyzed according to published methods [80,81], with minor adjustments. In brief, 5 ± 0.05 g of homogenized shellfish samples were extracted with 5 mL of an aqueous 1% acetic acid solution and vortexed for 90 s. The above-formed liquid was collected, boiled at 100 °C for 5 min, cooled in cold water until it reached room temperature, and centrifuged for 10 min at 4500 rpm. The supernatant (1 mL) obtained was removed, mixed with 5 μL of ammonium hydroxide, and centrifuged again for 10 min at 10,000 rpm for PST extraction using solid-phase extraction purification. An ENVI-Carb solid-phase extraction column was activated with 3 mL of acetonitrile, 3 mL of a 20% acetonitrile aqueous solution (containing 1% acetic acid), and 3 mL of 0.1% ammonium hydroxide. After activation, 500 μL of the supernatant was added to the column and 700 μL of ultrapure water was used for washing. Finally, 1 mL of a 75% acetonitrile solution (containing 0.25% formic acid) was employed for elution. All the extracts were filtered through a 0.22 μm membrane filter prior to the LC-MS/MS analysis.

### 4.4. LC-MS/MS Analysis

An LC-MS/MS analysis of the PST extracts was performed using an LC system (LC20ADXR, Shimadzu, Kyoto, Japan) coupled with a mass spectrometer (5500 QTRAP, Sciex, Framingham, MA, USA) equipped with Turbo V source and electrospray ionization (ESI) probe. In brief, a hydrophilic column (TSK-Amide-80, 150 mm × 2.0 mm, 3 μm) was held at 40 °C and a flow rate of 0.35 mL min^−1^, and used for the LC separation of the PSTs. The A and B mobile phases consisted of water and 95% acetonitrile in water, respectively, both containing 2 mM ammonium formate and 50 mM formic acid. The elution gradient was as follows: 80% B, held for 3 min; linear gradient to 40% B for 5 min and held for 2 min; and then back to 80% B within 1 min and held for 2 min [80,81]. The LC-MS/MS conditions are listed in Table S2, and the other parameters are provided in Table S3. In addition, the linear ranges and LODs of the 13 PSTs are provided in Table S4. The shellfish matrix absolute recovery of all target analytes was in the range of 82.5–92.2%. The precision of the LC-MS/MS method was less than 15%.

### 4.5. Dietary Exposure Assessment

The shellfish consumption data for the different age groups were obtained from the 5th Chinese Total Diet Study and the monitoring report on the nutrition and health status of Chinese residents (Table S5) [82,83]. The risks of acute dietary exposure to PSTs in the diets of consumers of different genders and ages were calculated as follows [84,85]:

$$\mathrm{PST}\;\mathrm{dietary}\;\mathrm{exposure}\;(\mu g\;{\mathrm{kg}}^{-1}\;\mathrm{bw}\;{\mathrm{day}}^{-1})\;=\frac{\mathrm{Toxicity}\;\mathrm{of}\;\mathrm{PSTs}\;\mathrm{in}\;\mathrm{shellfish}\;(\mu g\;\mathrm{STX}\;\mathrm{eq}.{\mathrm{kg}}^{-1})\;\times \;Shell\mathrm{fish}\;\mathrm{consumption}(\mathrm{kg}\cdot {day}^{-1})}{\mathrm{Body}\;\mathrm{weight}\;(\mathrm{kg})}$$

### 4.6. PST Toxicity Data

The toxicity of the PSTs in each shellfish sample was calculated by setting the toxicity factor (TEF) for the STX toxin to 1 and determining the TEF for each of the other toxins according to their toxicity relative to STX. The toxicity of PSTs in the samples was expressed according to the TEF (Table S6), converted uniformly to the STX equation, and the toxicity of the toxins in the shellfish samples was determined as the sum of the analogs of the PSTs. The FAO/IOC/WHO (2004) scheme for assigning the toxicity values to non-detects (NDs) was used in this study [23]. In the lower bound (LB) scenario, 0 was applied as a substitute for ND results [76,86], while in the upper bound (UB) scenario, the limit of detection (LOD) was employed as a substitute for the ND results. Shellfish samples with PSTs > 0 in the LB were defined as positive shellfish. All the positive shellfish samples were analyzed for differences in the composition of PSTs in different shellfish and for seasonal variations in PST toxicity. In the UB scenario, shellfish samples with maximum PST levels were used for the dietary exposure risk assessment. 

### 4.7. Statistical Analysis

A statistical analysis of all the data was performed with Microsoft Excel 2016, and statistical significance was evaluated through a one-way ANOVA with *p* < 0.05 using SPSS 22.0 (IBM Corporation, Armonk, NY, USA). Origin 2021 was employed for constructing graphs and ArcGIS 10.8 was used for spatiotemporal toxin distribution mapping.

## 5. Conclusions

The toxicity and profile of 13 PSTs in different shellfish along the Chinese coast were analyzed through LC-MS/MS. The results showed significant differences in the accumulation and major analogs of PSTs in different shellfish, as well as obvious seasonal variations in PST contamination along the Chinese coast. PST contamination was higher in spring, and the PST detection rate and toxicity in shellfish in Hebei Province were significantly higher than those in other provinces. Notably, mussels were more severely contaminated with PSTs during spring, posing a risk to consumers. However, none of the shellfish samples contained PSTs above the EU safety limit (800 µg STX eq. kg^−1^). Furthermore, susceptible populations such as children and the elderly were found to present relatively high dietary exposure risk. These findings suggested that it is crucial to strengthen the efforts to detect PST contamination in China’s coastal areas to improve consumer dietary safety.

## Figures and Tables

**Table 1:** Toxicities of PSTs in different shellfish.

Groups	Number of Analogs ^1^	Detection Rate ^2^ (%)	Average (µg STX eq. kg^−1^)		Max (µg STX eq. kg^−1^)	
			LB ^3^	UB ^4^	LB	UB
Clams	10	16.04	3.70	12.89	125.83	173.78
Oysters	7	22.50	12.67	24.04	70.39	117.92
Mussels	11	64.67	22.51	56.25	546.09	576.02
Scallops	12	81.48	15.95	61.72	133.32	161.72
Ark shells	11	75.00	62.23	94.67	233.20	247.20
Total	13	37.60	11.86	32.49	546.09	576.02

^1^: Number of analog of PSTs detected in the samples. ^2^: Percentage of shellfish with at least one analog of PSTs above its detection limit. ^3^: Lower bound. ^4^: Upper bound. The EFSA has so far mainly used substitution methods, i.e., for results below the LOD, a value equal to the LOD (upper bound), zero (lower bound), or half the LOD (medium or middle bound) is commonly used.

**Table 2:** Acute dietary exposure of consumers of different age groups to PSTs in shellfish.

Age	Gender	EDI (µg STX eq. kg^−1^ bw day^−1^)				
		Clams	Oysters	Mussels	Scallops	Ark Shells
2~7	male/female	0.23	0.16	0.78	0.22	0.33
8~12	male/female	0.18	0.13	0.60	0.17	0.26
13~19	male	0.17	0.12	0.57	0.16	0.24
	female	0.14	0.10	0.48	0.13	0.20
20~50	male	0.19	0.13	0.63	0.18	0.27
	female	0.19	0.13	0.62	0.17	0.27
51~65	male	0.20	0.14	0.67	0.19	0.29
	female	0.19	0.13	0.62	0.17	0.27
>65	male	0.23	0.16	0.77	0.22	0.33
	female	0.19	0.13	0.64	0.18	0.27

## Data Availability

Data will be made available on request.

## Supplementary Materials

The following supporting information can be downloaded at https://www.mdpi.com/article/10.3390/md22020064/s1: Table S1: Classification and detailed structure of PSTs; Table S2: LC–MS/MS conditions; Table S3: Mass spectrometric analysis parameters of 13 PSTs; Table S4: The linear range and LOD of 13 PSTs; Table S5: Body weight and consumption of bivalve shellfish in different age groups; Table S6: The toxic factor of PSTs.

## References

[1] McCarthy M., Bane V., García-Altares M., Pelt F., Furey A., O’Halloran J. (2015). Assessment of emerging biotoxins (pinnatoxin G and spirolides) at Europe’s first marine reserve: Lough Hyne. Toxicon.

[2] Huang Y. (2020). Analysis on the construction of traceability system for the quality and safety of aquatic products in Fujian Province. Fujian Agric. Sci. Technol..

[3] Lukowski A.L., Denomme N., Hinze M.E., Hall S., Isom L.L., Narayan A.R.H. (2019). Biocatalytic detoxification of paralytic shellfish toxins. ACS Chem. Biol..

[4] Etheridge S.M. (2010). Paralytic shellfish poisoning: Seafood safety and human health perspectives. Toxicon.

[5] Brown A.R., Lilley M., Shutler J., Lowe C., Artioli Y., Torres R., Berdalet E., Tyler C.R. (2020). Assessing risks and mitigating impacts of harmful algal blooms on mariculture and marine fisheries. Rev. Aquac..

[6] Wang D.Z., Zhang S.F., Zhang Y., Lin L. (2016). Paralytic shellfish toxin biosynthesis in cyanobacteria and dinoflagellates: A molecular overview. J. Proteom..

[7] Kosatsky T. (2021). Changing trends in paralytic shellfish poisonings reflect increasing sea surface temperatures and practices of indigenous and recreational harvesters in British Columbia, Canada. Mar. Drugs.

[8] Carvalho I.L.d., Pelerito A., Ribeiro I., Cordeiro R., Núncio M.S., Vale P. (2019). Paralytic shellfish poisoning due to ingestion of contaminated mussels: A 2018 case report in Caparica (Portugal). Toxicon X.

[9] Huang H.N., Lu J.L., Lin S.E., Zheng R.J., Lin J. (2020). Simultaneous determination of twelve paralytic shellfish poisoning toxins in bivalve molluscs by UPLC-MS/MS and its applications to a food poisoning incident. Toxicon.

[10] Llewellyn L.E. (2006). Saxitoxin, a toxic marine natural product that targets a multitude of receptors. Nat. Prod. Rep..

[11] Catterall W.A., Cestele S., Yarov-Yarovoy V., Yu F.H., Konoki K., Scheuer T. (2007). Voltage-gated ion channels and gating modifier toxins. Toxicon.

[12] Peacock M.B., Gibble C.M., Senn D.B., Cloern J.E., Kudela R.M. (2018). Blurred lines: Multiple freshwater and marine algal toxins at the land-sea interface of San Francisco Bay, California. Harmful Algae.

[13] Visciano P., Schirone M., Berti M., Milandri A., Tofalo R., Suzzi G. (2016). Marine Biotoxins: Occurrence, toxicity, regulatory limits and reference methods. Front. Microbiol..

[14] Shin C., Jo H., Kim S.H., Kang G.J. (2018). Exposure assessment to paralytic shellfish toxins through the shellfish consumption in Korea. Food Res. Int..

[15] Li Z.J., Chen J.H., Wang S., Zhang R.T., Shi Q., Zheng L., Wang X.R. (2014). MS characteristic of paralytic shellfish poisoning toxins detected by electrospray lonization mass spectrometry in negative ion mode. J. Chin. Mass. Spectrom. Soc..

[16] Yu R., Luo X. (2016). Status and research perspectives on toxic algae and phycotoxinsin the coastal waters of China. Stud. Mar. Sin..

[17] Hu X.L., Cheng J.G., Zhang G. (2012). Fast determination of paralytic shellfish poison (PSP) in Fresh Scallop by ELISA. Mod. Prev. Med..

[18] Qiu J.B., Meng F.P., Ding L., Che Y.J., McCarron P., Beach D.G., Li A.F. (2018). Dynamics of paralytic shellfish toxins and their metabolites during timecourse exposure of scallops *Chlamys farreri* and mussels *Mytilus galloprovincialis* to *Alexandrium pacificum*. Aquat. Toxicol..

[19] Du K., Jiang T., Wu N. (2013). The pattern of paralytic shellfish poisoning in shellfish cultured in the coast of Yellow Sea, China. Mar. Environ. Sci..

[20] Vale P., Taleb H. (2005). Assessment of the quantitative determination of paralytic shellfish poisoning toxins by pre-column derivatization and elimination of interfering compounds by solid-phase extraction. Food Addit. Contam..

[21] Alexander J., Benford D., Boobis A., Ceccatelli S., Cravedi J.P., Domenico A.D., Doerge D., Dogliotti E., Edler L., Farmer P. (2009). Marine biotoxins in shellfish–summary on regulated marine biotoxins scientific opinion of the panel on contaminants in the food chain. EFSA J..

[22] Chain E. (2009). Marine biotoxins in shellfish-Saxitoxin group. EFSA J..

[23] Andersen P., Aune T., Baden D., Belin C., Botana L.M., Busby P., Dickey B., Fessard V., Fleming L.E., Foorde J. Report of the joint FAO/IOC/WHO ad hoc expert consultation on biotoxins in bivalve molluscs. Proceedings of the Joint FAO/IOC/WHO ad hoc Expert Consultation on Biotoxins in Bivalve Molluscs.

[24] Zhou J. (2022). Development path and technological changes of China’s marine shellfish industry. Chin. Fish. Econ..

[25] Fishery Bureau of the Ministry of Agriculture and Rural Affairs of China (2023). China Fishery Statistical Yearbook.

[26] Li P.P. (2014). The analysis of nutrient ingredients and protein quality on five economic shellfish. Food Res. Dev..

[27] Liang Y.B., Li D.M., Yao J.Y., Jin W., Gong C.B., Liu R.Y. (2019). Progresses in investigation and research on phycotoxins and toxicmicroalgaes in the coastal waters of China. Oceanol. Limnol. Sin..

[28] Genovesi B., Berrebi P., Nagai S., Reynaud N., Wang J., Masseret E. (2015). Geographic structure evidenced in the toxic dinoflagellate *Alexandrium pacificum* Litaker (*A. catenella*—Group IV (Whedon & Kofoid) Balech) along Japanese and Chinese coastal waters. Mar. Pollut. Bull..

[29] Gao Y., Yu R.C., Chen J.H., Zhang Q.C., Kong F.Z., Zhou M.J. (2015). Distribution of *Alexandrium fundyense* and *A. pacificum* (Dinophyceae) in the Yellow Sea and Bohai Sea. Mar. Pollut. Bull..

[30] Gao Y., Yu R.C., Murray S.A., Chen J.H., Kang Z.J., Zhang Q.C., Kong F.Z., Zhou M.J. (2015). High specificity of a quantitative PCR assay targeting a saxitoxin gene for monitoring toxic algae associated with paralytic shellfish toxins in the Yellow Sea. Appl. Environ. Microbiol..

[31] Weng Q., Zhou B. (2023). Paralytic shellfish poisoning contamination status and dietary exposure assessment in coastal cities of China. Prev. Med..

[32] Liu Y., Yu R.C., Kong F.Z., Chen Z.F., Dai L., Gao Y., Zhang Q.C., Wang Y.F., Yan T., Zhou M.J. (2017). Paralytic shellfish toxins in phytoplankton and shellfish samples collected from the Bohai Sea, China. Mar. Pollut. Bull..

[33] Yu R.C., Zhang Q.C., Liu Y., Chen Z.F., Geng H.X., Dai L., Lin Z.R., Tang W.J., Kong F.Z., Yan T. (2021). The dinoflagellate producing only carbamate toxins may account for the seafood poisonings in Qinhuangdao, China. Harmful Algae.

[34] Lin X.T., Zhang M.S., Wang Z.J., Zhang Y.Y. (2005). Analysis of poisoning characteristics of paralytic shellfish poison in Haizhou Bay of Lianyungang, Jiangsu Province. Chin. J. Food Hyg..

[35] Chen J.Z., Hong S.P., Cai M.R., Chen L., Zhang T.L. (2018). Investigation of outbreaks of foodborne diseases caused by paralytic shellfish poisoning. Chin. J. Food Hyg..

[36] Shi W.B., Chen Y.P., Han X.Q., Wang Y.N., Li Q.C., Gao L.N. (2021). Dietary exposure assessment of 13 paralytic shellfish toxins in shellfish products sold in Tianjin. Jiangxi Fish. Sci. Technol..

[37] Zheng X.Y., Li Z.X., Sun X.H., Xie L.H., Zhang M.T., Zhu P.P., Wang J.Y., Su W.Q. (2023). Surveillance and risk assessment of diarrhetic and paralytic shellfish toxins in the Tangshan shellfish culture areas of Bohai Sea, China. Prog. Fish. Sci..

[38] Li C., Xiao W.L., Ye H.M., Lai X.C., Shi H., He C.H. (2023). Analysis of the pollution status of paralytic shellfish poisons in shellfish sold in Hainan Province, 2018-2021. China Trop. Med..

[39] Chorus I., Welker M. (2021). Toxic Cyanobacteria in Water: A Guide to Their Public Health Consequences, Monitoring and Management.

[40] Shahmohamadloo R.S., Frenken T., Rudman S.M., Ibelings B.W., Trainer V.L. (2023). Diseases and Disorders in Fish Due to Harmful Algal Blooms.

[41] Sekiguchi K., Sato S., Kaga S., Ogata T., Kodama M. (2001). Accumulation of paralytic shellfish poisoning toxins in bivalves and an ascidian fed on *Alexandrium tamarense* cells. Fish. Sci..

[42] Mizuta M., Yamada K., Takata K., Shimaoka M., Takayama H., Ouchi A. (1999). Differences of accumulation and elimination of paralytic shellfish poisons among oyster, mussel and scallop. Food Hyg. Saf. Sci. (Shokuhin Eiseigaku Zasshi).

[43] Braga A.C., Camacho C., Marques A., Gago-Martínez A., Pacheco M., Costa P.R. (2018). Combined effects of warming and acidification on accumulation and elimination dynamics of paralytic shellfish toxins in mussels *Mytilus galloprovincialis*. Environ. Res..

[44] Turner A.D., Hatfield R.G., Maskrey B.H., Algoet M., Lawrence J.F. (2019). Evaluation of the new European Union reference method for paralytic shellfish toxins in shellfish: A review of twelve years regulatory monitoring using pre-column oxidation LC-FLD. TrAC Trends Anal. Chem..

[45] Xu D.Y., Liu L., Yu J., Liu R.Y., Liang Y.B., Cheng X.M. (2013). Depuration of paralytic shellfish poisoning by laboratory in *Mytilus edulis*. Mar. Environ. Sci..

[46] Shumway S.E., Cucci T.L. (1987). The effects of the toxic dinoflagellate *Protogonyaulax tamarensis* on the feeding and behaviour of bivalve molluscs. Aquat. Toxicol..

[47] Xie W.C., Liu X.L., Yang X.H., Zhang C.H., Bian Z.Y. (2013). Accumulation and depuration of paralytic shellfish poisoning toxins in the oyster *Ostrea rivularis* Gould-Chitosan facilitates the toxin depuration. Food Control.

[48] Turnbull A., Dorantes-Aranda J.J., Madigan T., Jolley J., Revill H., Harwood T., Hallegraeff G. (2021). Field validation of the southern rock lobster paralytic shellfish toxin monitoring program in Tasmania, Australia. Mar. Drugs.

[49] Lassus P., Fremy J.M., Ledoux M., Bardouil M., Bohec M. (1989). Patterns of experimental contamination by *Protogonyaulax tamarensis* in some French commercial shellfish. Toxicon.

[50] Bao Z.M., Kong L.L., Shi J.X., Li M.L., Lian S.S., Wang H.Z., Wei Z.C., Hu J.J., Hu X.L. (2021). Research progress on accumulation and transformation of paralytic shellfish toxins in bivalves. Period. Ocean. Univ. China.

[51] Li S.C., Wang W.X., Hsieh D.P.H. (2001). Feeding and absorption of the toxic dinoflagellate *Alexandrium tamarense* by two marine bivalves from the South China Sea. Mar. Biol..

[52] Núñez-Acuña G., Aballay A.E., Hégaret H., Astuya A.P., Gallardo-Escárate C. (2013). Transcriptional responses of *Mytilus chilensis* exposed in vivo to saxitoxin (STX). J. Molluscan Stud..

[53] Detree C., Núñez-Acuña G., Roberts S., Gallardo-Escarate C. (2016). Uncovering the complex transcriptome response of *Mytilus chilensis* against saxitoxin: Implications of harmful algal blooms on mussel populations. PLoS ONE.

[54] Qiu J. (2014). Metabolic Transformation of Paralytic Shellfish Toxins by Bivalve Molluscs and Their Physiological and Biochemical Responses. Master’s Thesis.

[55] Gu H., Zeng N., Liu T., Yang W., Müller A., Krock B. (2013). Morphology, toxicity, and phylogeny of *Alexandrium* (Dinophyceae) species along the coast of China. Harmful Algae.

[56] Kim H.Y., Shin I.S. (2015). Comparison of paralytic shellfish toxin profiles of *Alexandrium tamarense* and blue mussel (*Mytilus edulis*) in Korea. Food Sci. Biotechnol..

[57] Suzuki T., Ichimi K., Oshima Y., Kamiyama T. (2003). Paralytic shellfish poisoning (PSP) toxin profiles and short-term detoxification kinetics in mussels *Mytilus galloprovincialis* fed with the toxic dinoflagellate *Alexandrium tamarense*. Harmful Algae.

[58] Hu X.Q., Jie W.C., Li M., Dong Z.Q., Yang X.H. (2021). Review on accumulation and metabolism of paralytic shellfish toxins in shellfish. Food Mach..

[59] Song W.J., Song X.X., Yu Z.M., Li J., Zhang Y. (2022). Research progress on the biosynthesis, transformation, and factors affecting paralytic shellfish toxins in the ocean. Mar. Sci..

[60] Asakawa M., Miyazawa K., Takayama H., Noguchi T. (1995). Dinoflagellate *Alexandrium tamarense* as the source of paralytic shellfish poison (PSP) contained in bivalves from Hiroshima Bay, Hiroshima Prefecture, Japan. Toxicon.

[61] Lin H.P., Cho Y., Yashiro H., Yamada T., Oshima Y. (2004). Purification and characterization of paralytic shellfish toxin transforming enzyme from *Mactra chinensis*. Toxicon.

[62] Zhang H., Zhang J. (2003). Toxic effects of paralytic shellfish poisonings and analytical techniques. Mar. Environ. Sci..

[63] Kotaki Y. (1989). Screening of bacteria which convert gonyautoxin 2, 3 to saxitoxin. Nippon Suisan Gakkaishi.

[64] Andres J.K., Yñiguez A.T., Maister J.M., Turner A.D., Olano D.E.B., Mendoza J., Salvador-Reyes L., Azanza R.V. (2019). Paralytic shellfish toxin uptake, assimilation, depuration, and transformation in the Southeast Asian Green-Lipped Mussel (*Perna viridis*). Toxins.

[65] Harley J.R., Lanphier K., Kennedy E., Whitehead C., Bidlack A. (2020). Random forest classification to determine environmental drivers and forecast paralytic shellfish toxins in Southeast Alaska with high temporal resolution. Harmful Algae.

[66] Shinada A. (2005). Limiting factor for growth of *Alexandrium tamarense* in the coastal water, northeastern part of Hokkaido, Japan in summer. Sci. Rep. Hokkaido Fish. Exp. Stn..

[67] Bao X.W., Li N., Yao Z.G., Wu D.X. (2009). Seasonal variation characteristics of temperature and salinity of the North Yellow Sea. Period. Ocean. Univ. China.

[68] Wang X.Z., Wu H., Cheng Y., Wen H.M., Liu R., Wang L.B., Shan C.X., Chai C. (2019). Multi-year assessment of paralytic shellfish toxins in hard clam species along the coastline of Jiangsu Province, China. Acta Oceanol. Sin..

[69] Yuan B.K., Huang R., Shang J., Jiao Y., Li J., Guo D.L., Jiang W.F. (2015). Coastal waters of Bohai Sea based on shore-based observation data. Ocean. Dev. Manag..

[70] Tobin E.D., Wallace C.L., Crumpton C., Johnson G., Eckert G.L. (2019). Environmental drivers of paralytic shellfish toxin producing *Alexandrium catenella* blooms in a fjord system of northern Southeast Alaska. Harmful Algae.

[71] Wang P., Mao K.B., Meng F., Yuan Z.J. (2020). Spatiotemporal evolution of sea surface temperature in the East China Sea. Remote Sens. Nat. Resour..

[72] Wang Z.F., Zhang Q., Lv H.Y. (2001). Effects of temperature, salintty, light and pH on the growth of red tide organisms *Prorocentrum micans*. Oceanol. Limnol. Sin..

[73] Yang M.H., Chen S.L., Wu J.W., Wu J., Long L.J., Zhou W.H. (2007). Research progress on marine *Prorocentrum dinoflagellates*. Mod. Chin. Med..

[74] Finnis S., Krstic N., McIntyre L., Nelson T.A., Henderson S.B. (2017). Spatiotemporal patterns of paralytic shellfish toxins and their relationships with environmental variables in British Columbia, Canada from 2002 to 2012. Environ Res.

[75] Wang H., Hu Z.X., Shang L.X., Leaw C.P., Lim P.T., Tang Y.Z. (2020). Toxicity comparison among four strains of *Margalefidinium polykrikoides* from China, Malaysia, and USA (belonging to two ribotypes) and possible implications. J. Exp. Mar. Biol. Ecol..

[76] Zhou Y., Li S., Zhang J., Zhang J., Wang Z., Pan L., Huang B., Huang K., Chen X., Zhao Q. (2022). Dietary exposure assessment of paralytic shellfish toxins through shellfish consumption in Shenzhen population, China. Environ. Sci. Pollut. Res. Int..

[77] Jones J.I.W., Hawkey C.J. (2001). Physiology and organ-related pathology of the elderly: Stomach ulcers. Best Pract. Res. Clin. Gastroenterol..

[78] Cao Y., Qiu J., Li A., Zhang L., Yan G., Ji Y., Zhang J., Zhao P., Wu X. (2023). Occurrence and spatial distribution of paralytic shellfish toxins in seawater and marine organisms in the coastal waters of Qinhuangdao, China. Chemosphere.

[79] Wu X., Gao M., Wang L., Luo Y., Bi R., Li L., Xie L. (2014). The arsenic content in marketed seafood and associated health risks for the residents of Shandong, China. Ecotoxicol. Environ. Saf..

[80] Wu H.Y., Dong C.F., Zheng G.C., Zhang Z.H., Zhang Y.Y., Tan Z.J., Gu H.F. (2022). Formation mechanism and environmental drivers of *Alexandrium catenella* bloom events in the coastal waters of Qinhuangdao, China. Environ. Pollut..

[81] Dong C.F., Wu H.Y., Peng J.X., Guo M.M., Zhai Y.X., Tan Z.J. (2022). Thermal processing induced release and degradation of paralytic shellfish toxin from mussels *Mytilus edulis*. J. Oceanol. Limnol..

[82] Wu Y.N., Zhao Y.F., Li J.G. (2018). The Fifth China Total Diet Study.

[83] Piao J.H., Huo J.S. (2019). The Monitoring Report on Nutrition and Health Status of Chinese Residents (2010–2013) (II).

[84] Wong W.W., Chung S.W., Chan B.T., Ho Y.Y., Xiao Y. (2013). Dietary exposure to inorganic arsenic of the Hong Kong population: Results of the first Hong Kong total diet study. Food Chem. Toxicol..

[85] Mbabazi J. (2011). Principles and methods for the risk assessment of chemicals in food. Int. J. Environ. Stud..

[86] EFSA, European Food Safety Authority (2010). Management of left-censored data in dietary exposure assessment of chemical substances. EFSA J..

