# Supplementary material for: Contamination Status and Risk Assessment of Paralytic Shellfish Toxins in Shellfish along the Coastal Areas of China
Source: Mar Drugs. 2024 Jan 26;22(2):64. doi: 10.3390/md22020064 (PMC10890588; doi:10.3390/md22020064)
Supplement: Supplementary file 1 [file marinedrugs-22-00064-s001.zip › marinedrugs-2764788-supplementary.pdf]

Table S1. Classification and detailed structure of PSTs.

Classification	Profile	R1	R2	R3	R4
carbamate toxins	STX	-H	-H	-H	$-OCONH_2$
	NEO	-OH	-H	-H	
	GTX1	-OH	$-OSO_3^-$	-H	
	GTX2	-H	$-OSO_3^-$	-H	
	GTX3	-H	-H	$-OSO_3^-$	
	GTX4	-OH	-H	$-OSO_3^-$	
n-sulfocarbamoyl toxins	C1	-H	$-OSO_3^-$	-H	$-OCONHSO_3^-$
	C2	-H	-H	$-OSO_3^-$	
	C3	-OH	$-OSO_3^-$	-H	
	C4	-OH	-H	$-OSO_3^-$	
	GTX5	-H	-H	-H	
	GTX6	-OH	-H	-H	
decarbamoyl toxins	dcGTX1	-OH	$-OSO_3^-$	-H	-OH
	dcGTX2	-H	$-OSO_3^-$	-H	
	dcGTX3	-H	-H	$-OSO_3^-$	
	dcGTX4	-OH	-H	$-OSO_3^-$	
	dcSTX	-H	-H	-H	
deoxydecarbamoyl toxins	doSTX	-H	-H	-H	-H
	doGTX2	-H	-H	$-OSO_3^-$	
	doGTX3	-H	$-OSO_3^-$	-H	

Table S2. LC-MS/MS conditions [82].

Liquid chromatography conditions		Mass spectrometry conditions	
chromatographic column	TSK-Amide-80（3μm, 2mm×150mm）	electrospray ionization	ESI
column temperature	40°C	Multi-response monitoring	MRM
flow velocity	0.35mL/min	mode	positive and negative ion switching mode
sample volume	5μL		
move phase A	water (containing 2 mmol/L ammonium formate, 50 mmol/L formic acid)	spray voltage	5.5kV, –4.5kV
move phase B	95% aqueous acetonitrile solution (containing 2 mmol/L ammonium formate, 50 mmol/L formic acid)	ion source temperature	550°C
elution gradient	3.0min, 80% B	crash gas pressure	Medium
	5.0min, 40% B	air curtain pressure	30psi
	2.0 min, 40% B	atomizing gas pressure GS1	50psi
	2.0min, 80% B	auxiliary heating gas pressure GS2	50psi

Table S3. Mass spectrometric analysis parameters of 13 PSTs.

Compound	Retention time	Parent ion	Qualitative and quantitative ions	Collisional energy	Deculturization voltage	Selection ion mode
STX	6.11	300.2	221.0/204.0	35/30	30	Positive ions
dcSTX	6.11	257.1	239.1/126.1	22/30	30	Positive ions
NEO	6.14	316.1	298.2/126.1	34/34	34	Positive ions
dcNEO	6.07	273.1	225.2/126.1	35/35	35	Positive ions
GTX5	6.09	380.1	300.1/282.1	15/35	25	Positive ions
GTX2	5.92	394.0	333.1/351.1	-22/16	16	Negative ions
GTX3	6.11	394.0	351.1/333.1	-16/22	22	Negative ions
GTX1	5.95	410.1	349.1/367.1	-22/15	15	Negative ions
GTX4	6.14	410.1	367.1/349.4	-15/22	22	Negative ions
C1	5.85	474.1	122.0/351.1	-25/30	30	Negative ions
C2	6.06	474.1	122.0/351.1	-30/25	25	Negative ions
dcGTX2	5.99	351.1	333.1/164.0	-17/30	30	Negative ions
dcGTX3	6.17	351.1	164.0/333.1	-30/17	17	Negative ions

Table S4. The linear range and LOD of 13 PSTs.

PST	Linear range(ng/mL)	LOD(μg/kg)
STX	5.0-500	10.0
dcSTX	5.0-500	10.0
NEO	5.0-500	10.0
dcNEO	5.0-500	10.0
GTX5	5.0-500	10.0
GTX2	4.67-467	12.0
GTX3	2.00-200	14.0
GTX1	6.35-635	12.0
GTX4	2.00-200	4.0
C1	6.74-674	13.0
C2	2.00-200	4.0
dcGTX2	6.88-688	14.0
dcGTX3	2.00-200	4.0

Table S5. Body weight and consumption of bivalve shellfish in different age groups [83-84].

Age	Gender	Average weight (kg)	Average daily consumption of shellfish (g)
2~7	male/female	16.2	21.8
8~12	male/female	35.9	37.3
13~19	male	55.1	54.1
	female	50.0	41.3
20~50	male	63.1	69.3
	female	55.7	60.1
51~65	male	64.4	74.5
	female	58.4	63.1
>65	male	61.1	81.6
	female	54.9	61.0

Table S6. The toxic factor of PSTs [77].

PST	TEF
STX	1
NEO	1
dcSTX	1
dcNEO	0.4
GTX1	0.4
GTX4	0.7
GTX2	0.4
GTX3	0.6
GTX5	0.1
dcGTX2	0.2
dcGTX3	0.4
C1	0.01
C2	0.1
